# Smart cost estimation: Empirical case for extra-high voltage transmission towers

**DOI:** 10.1016/j.heliyon.2024.e31466

**Published:** 2024-05-16

**Authors:** Diana Wahyu Hayati, Jieh-Haur Chen, Yu-Chun Chen, Shixian Li, Machsus Machsus, Mohamad Khoiri, Qian-Cheng Wang, Hsi-Hsien Wei

**Affiliations:** a Department of Civil Engineering, National Central University, Jhongli, 320317, Taoyuan, Taiwan; b Department of Civil Engineering, Research Center of Smart Construction, National Central University, Jhongli, 320317, Taoyuan, Taiwan; c Department of Transmission Line and Substation Projects, Taiwan Power Company, 10078, Taipei, Taiwan; d Department of Building and Real Estate, Hong Kong Polytechnic University, 999077, Hong Kong Special Administrative Region of China; e Department of Civil Infrastructure Engineering, Faculty of Vocations, Institut Teknologi Sepuluh Nopember, Indonesia; f Department of Land Economy, University of Cambridge, Cambridge, CB3 9EP, United Kingdom

**Keywords:** Energy, Extra-high voltage (EHV) transmission towers, Construction cost estimation, Support vector regression (SVR)

## Abstract

Nowadays, electricity has become an integral part of human lives. Most of our daily appliances, tools, and personal belongings are inseparable from electricity. To ensure a proper electricity distribution with an efficient transfer capability, Extra-High Voltage (EHV) transmission towers are needed. To design such a structure, it is of utmost importance to account for the cost of said tower. However, the process to estimate the cost of EHV transmission towers is both time-consuming and strenuous on human labor since a lot of consideration have to be taken. To overcome this, an imperative requirement exists for a prompt, precise, and automated tool to replace the existing manual cost estimation method. This research endeavor aims to craft a tool using support vector regression (SVR) with the capacity to prognosticate construction expenses for projects involving EHV transmission towers. The exploration of pertinent literature has enabled us to amass historical data and delineate the attributes essential for estimating costs linked to EHV transmission tower construction. The investigation delves into a comprehensive dataset spanning the past decade in Taiwan. Within this timeframe, 317 EHV transmission towers were erected between 2009 and 2019. However, 79 of these instances are excluded due to incomplete information, thereby yielding 238 viable datasets (comprising 75 % of the overall total) to underpin the development of the SVR model. By configuring the parameters to C = 0.2 and γ = 0.1, followed by 5-fold cross-validation, the resultant SVR model attains a remarkable prediction accuracy of 97.91 %, on average. As a result, the proposed SVR-based model can effectively and accurately predict the cost of constructing an EHV transmission tower project and reduce the time spent on estimation, thus contributing to the enhancement of the resilience and robustness of the transmission network system.

## 1 Introduction

Over the past decade, Taiwan has witnessed a significant surge in energy demands propelled by industrial progress. Highlighted in the revision to Taiwan's Electricity Act unveiled on January 11th, 2017, the Taiwanese government adheres to the guiding principle of "green energy first, diversified supply." This strategic approach aims to recalibrate the energy production landscape by elevating the prominence of renewable energy power plants. To this end, the Taiwan Power Co., Ltd. (referred to as Taipower), a state-owned entity responsible for electricity provision across Taiwan and its offshore island, aligns its operations with the government's directives outlined in these guidelines. In line with government aspirations, which aim to elevate the installed production capacity to 27 GW by 2025, capable of generating around 51.5 billion kWh of electricity, Taipower is progressively realigning its energy composition to accord with official energy policies. The company is achieving this by augmenting the contribution of renewable energy and incrementally enhancing the power supply within Taiwan. As part of these efforts, Taipower is spearheading an array of power transmission and transformation initiatives aimed at bolstering the resilience and robustness of the transmission network system. Presently, the foremost plan involves instituting an offshore wind power network in the northern region. Simultaneously, endeavors are underway in the central district, encompassing the erection and refurbishment of an extensive array of transmission towers spanning hundreds of kilometers, thereby laying the groundwork for an overarching extra-high voltage (EHV) transmission system. Additionally, Taipower has earmarked the replacement of EHV transmission towers that have been in operation for over four decades. This strategic shift anticipates the future requirement for the construction of numerous ultra-high voltage transmission towers. Constructing a singular EHV transmission tower constitutes a substantial undertaking marked by an extended project timeline and a multi-year budget. While uncompleted projects can carry over their budgets to subsequent years, real-world scenarios often lead to losses due to inflation-induced cost escalations. Should expenses surpass the initial budgetary allocations, projects could face postponement or even termination, necessitating further deliberation.

Commencing in 1997, the Taiwanese government embarked on a journey of refurbishing its EHV transmission towers. Yet, challenges emerged when it came to awarding construction contracts due to the low bids submitted for these projects. In order to sustain progress, budgets had to be augmented. Regrettably, the process of recalibrating these budgets to attain precision is intricate and time-intensive. What's urgently required is a swifter and more accurate approach to determine the construction costs for EHV transmission towers. Such a method should not only enhance the accuracy of cost assessment but also reduce the time spent on estimations. There is a pronounced need for an efficient, precise, and automated tool that can supersede the existing manual estimation process. The central aim of this study is to create a tool employing support vector regression (SVR) that possesses the capability to forecast construction expenses for EHV transmission tower projects.

## 2 Extra-high voltage transmission tower foundations

The provision of electricity stands as a pivotal facet of the national infrastructure, serving as a cornerstone for modern life and economic advancement. Taipower presently stands as Taiwan's sole integrated electric power company, assuming the responsibilities of power generation, transmission, distribution, and sales. The power grid in Taiwan can be categorized into three principal systems: power generation, transmission, and distribution. An intrinsic aspect of the transmission and power supply system is the network of transmission lines, encompassing both underground cable transmission lines and overhead transmission lines, each carrying varying voltage levels—namely, 345 kV, 161 kV, and 69 kV. The overhead transmission lines at the highest voltage level, 345 kV, are commonly referred to as Extra-High Voltage (EHV) transmission lines. Taipower's EHV overhead transmission lines can be dissected into three constituent parts: the tower foundation, iron towers, and transmission cables. Within the realm of foundations [[1], [2], [3], [4], [5]], three distinct types are discernible: Base foundations: Encompassing ordinary concrete foundations, and raft foundations. Pile foundations: Encompassing large diameter pile foundations (including reverse circulation type drilling concrete foundation, full casing type drilling concrete foundation), precast concrete foundation piles, and single foundation piles. Pier foundations: Encompassing caisson foundations, deep foundations, foundations for expansion, and single foundations. In the context of Taiwan's geographical characteristics, deep EHV transmission tower foundations find utility in terrains that are challenging for mechanical excavation, while single pile foundations are preferred for steep and rugged terrain. Caisson foundations are implemented in watery areas such as river valleys, or locations with substantial groundwater. The adoption of other foundation types is infrequent. The diverse foundational structures corresponding to the 238 tower foundations selected for this study are documented in Table 1.

**Table 1:** Basic types of EHV transmission tower foundation.

Basic type	Year									
	2010	2011	2012	2013	2014	2015	2016	2017	2018	2019 (Jan–March)
Deep foundation	9	21	24	27	2	0	0	0	10	1
Single pile	6	13	22	27	3	0	0	6	65	2
Caisson foundation	0	0	0	0	0	0	0	0	0	0
Other	0	0	0	0	0	0	0	0	0	0

## 3 SVR applications

The concept of support vectors was originally introduced by Russian statisticians Vapnik et al., in 1963. In 1974, Vapnik and Chervonenkis proposed the method of structural risk minimization (SRM), which subsequently paved the way for the advancement of statistical learning theory. To address non-linear classification, scholars suggested employing Kernel tricks in the construction of maximum margin hyperplanes [6]. The formulation of the support vector machine (SVM) followed a few years later [7]. In 1997, they extended their work by introducing the regression support vector machine model utilizing the *ε*-insensitivity loss function. This innovation expanded the breadth of SVM methods as growing numbers of researchers delved into this domain [8]. During the establishment of the Support Vector Regression (SVR) equation, deviations in outcomes may arise if the pertinent data isn't consistently appropriate for every case. To mitigate inaccuracies stemming from the regression model, a loss function and penalty parameter were introduced. The resultant linear SVR equation derived in this study is as follows [8]:

(1) $$f\left(x\right)=\sum_{i=1}^{m}\left({\alpha_{i}}^{*}-\alpha_{i}\right){x_{i}}^{T}x_{j}+b$$

$where\;b=y_{i}+\varepsilon -\sum_{i=1}^{m}\left({\alpha_{i}}^{*}-\alpha_{i}\right){x_{i}}^{T}x_{j}$ and ${\alpha_{i}}^{*}-\alpha_{i}\neq 0$.

Regarding non-linear issues, one approach involves employing a nonlinear mapping function φ (kernel function) to transition from the initial space to a feature-rich high-dimensional space, accomplished through the equation [8]:

(2) y=f(x)=(w·φ(x))+b

Converting the inherent non-linear data challenge into a higher-dimensional scenario has been explored in prior research. In instances where the problem exhibits linearity, SVR can be employed to forecast within the transformed space. The resolution of the decision function for non-linear problems entails [[9], [10], [11]]:

$$K\;\left(x,x_{i}\right)=\psi \left(x\right)‧\psi \left(x_{i}\right)$$

(3) $$f\left(x\right)=\sum_{i=1}^{m}\left(\alpha_{i}^{*}-\alpha_{i}\right)K\left(x,x_{i}\right)+b$$

In recent times, the domain of SVR forecasting techniques and technologies has garnered substantial attention in scholarly literature, showcasing achievements spanning a diverse array of fields. These include domains such as power generation, mechanical engineering, water conservation, finance, stock market prediction, medicine, chemistry, and biochemistry, among others. Across these applications, the results consistently underscore the robust predictive prowess of SVR. A noteworthy example emerged where a predictive model amalgamated chaotic mapping, the firefly algorithm, and SVR to forecast stock market prices [12]. This fusion of techniques demonstrated superior performance, surpassing other approaches like genetic algorithm-based SVR, chaotic genetic algorithm-based SVR, firefly-based SVR, artificial neural networks (ANNs), and adaptive neuro-fuzzy inference systems (ANFIS). For machine health monitoring and to predict short-term freeway traffic flow, an online SVR-based approach was introduced [13,14]. Furthermore, within the realm of project cost estimation, a study identified 14 influencing factors in the valuation of construction projects in Indonesia [15]. To enhance accuracy and efficiency in preliminary project valuation, the researchers constructed an SVR model. This model underwent testing on data from 104 building projects in Indonesia, showcasing a 95.79 % accuracy in engineering project estimations while significantly reducing evaluation time. Addressing the intricacies associated with predicting the purchase value of military aircraft, a devised SVR prediction model analyzed the impact of individual parameters, yielding heightened prediction accuracy [16]. Other than that, studies done by previous researchers showed that local optimal solutions can be obtained by SVR to increase its accuracy [17,18], and the method to solve the problem of nonlinear fitting has also been shown [19,20]. Moreover, other applications such as solving electric load forecasting [21] or constructing predictive models using the scikit-learn module have been shown using SVR [22,23]. Given its adeptness in a multitude of professional fields, as elucidated earlier, SVR stands out for its strong predictive capabilities. Moreover, due to its general superiority and stability compared to neural networks, SVR was selected as the preferred prediction method in the present research endeavor. This choice aims to formulate a prediction model for the cost of EHV transmission tower foundations supporting a transmission voltage of 345 kV.

## 4 Data collection and analysis

Taipower stands as the sole integrated power entity in Taiwan. The EHV transmission line tower project is administratively divided into three construction zones, each with its own office (North District, Central District, and Southern District), all operating within the Taipower Transmission and Transformation Project. Additionally, six district operation centers under the Power Supply Division (Taipei) - Xintao, Taichung, Jianan, Gaoping, and Huadong - are entrusted with overseeing project procurement and contracting activities. These responsibilities encompass pre-planning design, supervision post the final price agreement and related project management. The foundation of this study is rooted in data sourced from the government e-procurement network, spanning the past decade from January 2010 to March 2019. This specific 10-year timeframe was chosen due to its representative nature and the constraints of available e-procurement data. Limitations of data availability, along with the potential divergence of older data from current contexts due to inflation, justify this interval. Conversely, selecting a smaller time span could compromise the robustness of the results by lacking sufficient data points. From the construction data pertaining to EHV transmission lines over this 10-year period, 317 data sets were collected nationwide. However, 79 of these records contained incomplete information and were thus excluded, leaving 238 complete data sets for constructing the SVR model. Notably, Taipower has prioritized renewable energy development, exemplified by projects such as the Greater Changhua Offshore Wind Project slated to replace nuclear power generation in the northern region. The energy generated by this project necessitates distribution across the northern region via transmission lines, mandating the expansion of tower foundations and network infrastructure stretching from Taipei to Nantou. Consequently, a substantial portion of forthcoming tower foundation construction and renovation is concentrated in the northern and central regions of Taiwan. The data collected in this study offer insights into the locations of these tower foundations. With the exception of two locations in Tainan County, the remaining locations span seven counties including Taipei City, New Taipei City, Taoyuan City, Hsinchu City, Miaoli County, Taichung City, and Nantou County, aligning with the anticipated expansion projects' geographical scope. In-depth statistical analyses are conducted on the data encompassing each foundation type, location, procurement grade, construction period, procurement method, and date of the final price agreement.

1. Foundation Types: Among the existing EHV transmission towers, two foundation types have been identified: deep foundations and single pile foundations. The data analysis reveals a distribution of 94 instances (39 %) for the deep foundation type and 144 instances (61 %) for the single pile foundation type.
2. Project Locations: The geographic distribution of EHV transmission tower network construction includes cases in two locations in Tainan, twelve in Nantou, seven in Taichung, thirty-seven in Miaoli, twenty in Hsinchu, seventy-four in Taoyuan, and eighty-six in New Taipei City.
3. Procurement Levels: In adherence to government procurement regulations, the final price agreement cutoff is set at NT$50 million. The winning bids fall into distinct categories: less than 50 million (19 projects), 50–100 million (12 projects), 100–150 million (9 projects), and 150–200 million (6 projects).
4. Construction Periods: The construction periods are classified into four categories: less than one year (13 projects), one to two years (28 projects), two to three years (4 projects), and more than three years (1 project).
5. Date of Final Price Agreement: The dates of final price agreements on the e-procurement network are organized by year: 15 data sets in 2010, 34 in 2011, 46 in 2012, 54 in 2013, 5 in 2014, 6 in 2017, 75 in 2018, and 3 data sets in 2019.

Purchasing Method: In all instances included in this study, the procurement method utilized is open bidding, featuring a predetermined reserve price and subsequent contract negotiation based on the lowest bid.

6. The process of forecasting the fundamental cost of EHV Transmission tower foundations in this study unfolds through three distinct stages, which correspond to historical data segments: the initial budget, the reserve price, and the final price agreement. Xu's exploration of road engineering project pricing supports the feasibility of predicting the reserve price over the strike price [24]. Consequently, this study employs the base price for the EHV Transmission tower foundation, derived from the accumulated data, as the fundamental cost to be predicted. This cost is deemed the dependent variable (also known as the output variable, denoted as Y). Conversely, the independent variable (also known as the input variable, labeled as X), which encompasses several contributing factors, constitutes the basic cost of the EHV Transmission tower foundation.

Within this study, thirteen independent variables (X1-X13) are considered: deep foundation, single pile, rebar weight, concrete, manual excavation, clear water template, common template, basic transport weight, tower installation weight, steel plate retaining equipment fee (2 m ≤ diameter ≤3 m), steel plate retaining equipment fee (3.5 m ≤ diameter ≤4.5 m), steel ring retaining equipment fee (5 m ≤ diameter ≤6 m), and steel ring retaining equipment fee (6.5 m ≤ diameter ≤7.5 m), as outlined in Table 2. To accommodate the fluctuating costs of input construction materials over the ten-year span of data selection, price adjustments are warranted. These adjustments are computed using the "Construction Price Index" stipulated by the Taiwanese government in March 2019, as depicted in Equation (4).

(4) Adjustedreserveprice=Originalreserveprice×Constructionprojectpriceindex/constructionpriceindexatthetimewhentheoriginalreservepricewasmade

**Table 2:** Description of variable definition.

Variable Name	Variable Description	Unit
Deep foundation	Tower foundation type	seat
Single pile	Tower foundation type	seat
Rebar weight	The total weight of the steel bars used in the foundation of the tower	Tons
Concrete	Amount of concrete poured for the tower foundation	Cubic meters
Manual excavation	The amount of earthwork carried out using human resources	Cubic meters
Clear water template	The amount of material for shaping the concrete	Square meters
Common template	The amount of material for shaping the concrete	Square meters
Basic transport weight	Weight of steel, concrete, and other material moved to the construction site	Tons
Tower installation weight	Weight of iron components required to build the tower	Tons
Steel plate retaining equipment fee (2 m ≦ diameter ≦3 m)	Number of temporary facilities needed to support the pressure of the earth during the deep foundation excavation process	Square meters
Steel plate retaining equipment fee (3.5 m ≦ diameter ≦4.5 m)	Number of temporary facilities needed to support the pressure of the earth during deep foundation excavation process	Square meters
Steel ring retaining equipment fee (5 m ≦ diameter ≦6 m)	Number of temporary facilities needed to support the pressure of the earth during the excavation of a single pile	Square meters
Steel ring retaining equipment fee (6.5 m ≦ diameter ≦7.5 m)	Number of temporary facilities needed to support the pressure of the earth during the excavation of a single pile	Square meters

The scope of distribution spans a broad and intricate spectrum (refer to Table 3), necessitating standardization to enhance the precision of model predictions. In pursuit of this, zero-mean normalization is employed as the chosen standardization technique. This process transforms the data into a Gaussian distribution characterized by a mean of 0 and a variance of 1. This normalization approach ensures uniform data sizing, thus facilitating more effective model training. The formula is outlined below:

(5) $$x_{i}=\frac{x-\mu }{\sigma }$$

where $x_{i}$ is the converted data; x is the original value; μ is the sample value average; σ is the standard deviation of the sample data.

**Table 3:** Data basis analysis.

	Y	X1	X2	X3	X4	X5	X6
Quantity	238	238	238	238	238	238	238
Average Value	16,306,020	0.395	0.605	776.9877	882.376	284.4336	156.6992
Standard Deviation	3,417,137	0.2948	0.2948	197.1032	182.2705	102.4936	119.9156
Minimum Value	7,712,602	0	0	334.8367	439,33	0	0
Maximum Value	26,346,520	1	1	1265.5	1411	599	435
	**X7**	**X8**	**X9**	**X10**	**X11**	**X12**	**X13**
Quantity	238	238	238	238	238	238	238
Average Value	108.8993	2112.1865	52.7108	117.2092	181.1273	114.6676	192.8979
Standard Deviation	28.0176	441.9674	38.7211	161.3426	166.2346	154.1631	153.4312
Minimum Value	38.84	1068.1247	2.2	0	0	0	0
Maximum Value	210.15	3455.45	131.38	672	627.9	518.355	654

## 5 Construction of the tower foundation cost forecasting model

### 5.1 Development of the SVR prediction model

A total of 238 data points were gathered for this study, and subsequently subjected to standardization. These data were then randomly partitioned, resulting in 190 instances allocated for training and 48 instances designated for testing, maintaining an 8:2 ratio. The training data set is employed to train the predictive model, whereas the test data set serves to assess the overall performance of the fully trained model. The model's configuration involves the utilization of two parameters, specifically the cost parameter C and the width function γ, structured as follows:

C: [0.01,0.02,0.05,0.1,0.2,0.5,1,2,3,4,5,6,7,8,9,10,20,30,40,50];

γ: [0.001,0.002,0.005,0.01,0.02,0.05,0.1,0.2,0.5,1,2,3,4,5,6,7,8,9,10].

In this study, K-fold cross-validation with 5 folds is employed to gauge the prediction error of the SVR model. The application of 5 K-folds within K-fold cross-validation serves to counteract the potential pitfalls of overfitting or underfitting [25,26]. Furthermore, the grid search method is utilized to incorporate predefined parameters into the SVR model for 5-fold cross-validation training. The process unfolds as follows: the training data is initially divided into five equivalent sections. Subsequently, four of these sections are combined with specific parameters to train the model. Following training, the remaining data are utilized for verification, and accuracy scores are assessed. This process is iterated five times, with the verification data substituted in each repetition. The average of these five accuracy verification scores is then recorded. Different combinations of parameters are iteratively imported for training and verification until each parameter set completes the training and verification cycle. The results from individual cross-validations, along with the average test accuracy across different parameter combinations, are analyzed. The Python program and its integration of grid search and cross-validation technology are employed for calculations. The computed optimal values are determined to be C = 50 and γ = 0.05, utilizing the radial basis function (RBF) core function. These calculated optimal parameters yield the highest accuracy and the smallest standard deviation, indicating the effectiveness of this parameter combination in achieving the best prediction results within the SVR model. These values then form the basis for constructing the SVR prediction model, and the training outcomes are detailed in Table 4. In the quest to identify the best parameters, it's vital to comprehend the nuances in prediction model accuracy among various core functions by choosing the highest mean_test_score from all the prediction results. This study introduces both linear and polynomial (Poly) functions into the model for cross-validation training. Different parameter selections for various core functions yield diverse accuracy scores, influencing prediction performance. For the linear function, the optimal values are determined to be C = 0.2 and γ = 0.001, as indicated in Table 5. On the other hand, for the Poly function, optimal values are C = 0.2 and γ = 0.1, as shown in Table 6. While the optimal parameters for linear and Poly functions are lower compared to the RBF function for training and verification, it's corroborated that the RBF function stands as the most suitable choice for the SVR prediction model.

**Table 4:** Model training results (kernel = RBF).

Iteration No.	1816	1741	1594	1744	1669	1819	1591
param_C	50	40	20	40	30	50	20
param_gamma	0.05	0.05	0.1	0.1	0.1	0.1	0.05
param_kernel	rbf	rbf	rbf	rbf	rbf	rbf	rbf
split0_test_score	0.958444	0.953994	0.955198	0.956957	0.956865	0.956838	0.942019
split1_test_score	0.940674	0.93962	0.931098	0.929197	0.930546	0.927618	0.946728
split2_test_score	0.919844	0.913883	0.88877	0.931384	0.915509	0.936202	0.908021
split3_test_score	0.907531	0.891504	0.921987	0.872199	0.88492	0.867079	0.881461
split4_test_score	0.991774	0.991379	0.990927	0.990728	0.990827	0.990611	0.988032
mean_test_score	0.943653	0.938076	0.937596	0.936093	0.935733	0.935669	0.933252
std_test_score	0.029715	0.034209	0.034129	0.038917	0.036055	0.040587	0.036272
rank_test_score	1	2	3	4	5	6	7
split0_train_score	0.973944	0.973074	0.972852	0.973966	0.974052	0.97394	0.970181
split1_train_score	0.977549	0.976978	0.979119	0.980988	0.980576	0.981326	0.974387
split2_train_score	0.978529	0.97658	0.978326	0.979747	0.979491	0.978942	0.972234
split3_train_score	0.981752	0.978212	0.982844	0.981571	0.981545	0.982358	0.970107
split4_train_score	0.974413	0.973618	0.973076	0.975027	0.974241	0.975446	0.967081
mean_train_score	0.977238	0.975692	0.977243	0.97826	0.977981	0.978402	0.970798
std_train_score	0.002863	0.001997	0.003813	0.003147	0.003198	0.00326	0.002434

**Table 5:** Model training results (kernel = Linear).

Iteration No.	225	522	150	375	300	525	750
param_C	0.2	0.5	0.1	0.4	0.3	0.6	0.9
param_gamma	0.001	10	0.001	0.001	0.001	0.001	0.001
param_kernel	linear	linear	linear	linear	linear	linear	linear
split0_test_score	0.762776	0.74596	0.76512	0.751147	0.758514	0.738537	0.727362
split1_test_score	0.720447	0.720337	0.715492	0.720583	0.720485	0.720533	0.722728
split2_test_score	0.782039	0.792356	0.777035	0.786547	0.778611	0.792723	0.801987
split3_test_score	0.723612	0.720488	0.725837	0.722532	0.723084	0.71943	0.718649
split4_test_score	0.780089	0.785866	0.780925	0.782512	0.778389	0.787192	0.787277
mean_test_score	0.753793	0.753001	0.752882	0.752664	0.751817	0.751683	0.751601
std_test_score	0.026806	0.030992	0.027015	0.028208	0.025597	0.032027	0.035549
rank_test_score	384	409	434	460	485	510	535
split0_train_score	0.778165	0.784443	0.766333	0.782614	0.781964	0.785165	0.786848
split1_train_score	0.784419	0.784495	0.779893	0.784507	0.784767	0.784395	0.785744
split2_train_score	0.765879	0.780016	0.75972	0.776155	0.770336	0.780478	0.777366
split3_train_score	0.764135	0.763405	0.763167	0.764746	0.765041	0.763297	0.76291
split4_train_score	0.753627	0.7655	0.750117	0.760189	0.754951	0.767942	0.772169
mean_train_score	0.769245	0.775572	0.763846	0.773642	0.771412	0.776256	0.777007
std_train_score	0.010874	0.009247	0.009692	0.009644	0.010976	0.00895	0.008893

**Table 6:** Model training results (kernel = Poly).

Iteration No.	245	1664	917	26	1589	320	842
param_C	0.2	30	2	0.01	20	0.3	1
param_gamma	0.1	0.02	0.05	0.3	0.02	0.1	0.05
param_kernel	poly	poly	poly	poly	poly	poly	poly
split0_test_score	0.762212	0.771953	0.77461	0.779745	0.751501	0.78718	0.740807
split1_test_score	0.679857	0.691992	0.693319	0.698929	0.660772	0.705007	0.647684
split2_test_score	0.523554	0.479363	0.466626	0.440745	0.550259	0.390922	0.563049
split3_test_score	0.647283	0.654905	0.656855	0.660499	0.638851	0.665599	0.632288
split4_test_score	0.797913	0.811273	0.814419	0.820455	0.786936	0.827265	0.776175
mean_test_score	0.682164	0.681897	0.681166	0.680075	0.677664	0.675195	0.672001
std_test_score	0.096109	0.115498	0.121041	0.132407	0.084162	0.15327	0.076975
rank_test_score	1092	1093	1095	1096	1098	1099	1104
split0_train_score	0.744155	0.75841	0.76142	0.767286	0.728445	0.775729	0.710149
split1_train_score	0.742286	0.75168	0.753971	0.757945	0.73123	0.763338	0.718616
split2_train_score	0.772795	0.786751	0.790094	0.796856	0.754913	0.808065	0.74057
split3_train_score	0.762887	0.778435	0.782019	0.788545	0.74193	0.797832	0.724579
split4_train_score	0.717939	0.73471	0.73889	0.746977	0.702817	0.756838	0.688457
mean_train_score	0.748012	0.761997	0.765279	0.771522	0.731867	0.78036	0.716474
std_train_score	0.018908	0.018692	0.018629	0.018635	0.017253	0.019684	0.017181

### 5.2 Forecasting EHV transmission tower foundation costs

**Fig. 1:**
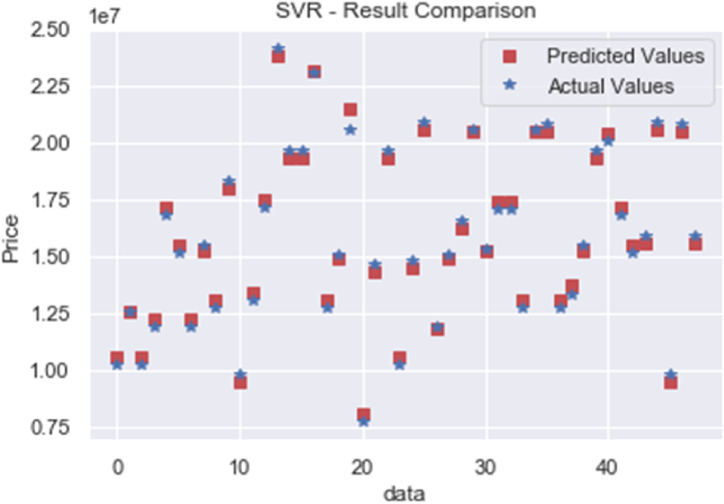
Comparison of SVR prediction results.

**Fig. 2:**
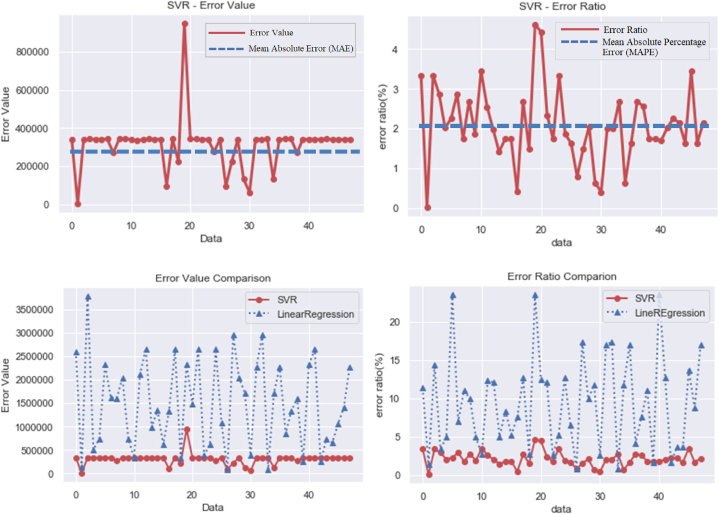
SVR Mean Absolute Error (MAE), Mean Absolute Percentage Error (MAPE), error rate comparison charts, and comparison of absolute values of errors.

The test dataset is input into the prediction model to estimate the tower foundation costs. The resulting predictions are then compared to the actual values, as depicted in Fig. 1. Examining the mean absolute error (MAE), the forecasted value and actual value discrepancy stands at 312,672 NTD, with the maximum error at 949,878 NTD and the minimum at 1533 NTD, as illustrated in Fig. 2. The mean absolute percentage error (MAPE) equates to 2.0916 %, with the maximum error rate at 4.6718 % and the minimum at 0.0122 %, again shown in Fig. 2. By gauging the prediction and actual data, the model's accuracy is calculated. Accounting for absolute value, the average accuracy emerges at a remarkable 97.9084 %, underscoring the model's exceptional predictive prowess. Full data are tabulated in Table 7. To establish a benchmark, comparisons are made with predictions derived from different methods. For instance, a predictive comparison model employing multiple linear regression is introduced, alongside predictions made under identical conditions using the Python program and the Scikit-learn suite, utilizing the same dataset of 238 instances. In the case of the multiple linear regression prediction outcomes, the MAE between forecasted and actual values is 1,490,349 NTD, with a maximum error of 3,856,561 NTD and a minimum error of 69,549 NTD. The MAPE calculates to 9.3426 %, with the maximum error rate at 24.553 % and the minimum at 0.6811 %. Once again, calculations using actual and predicted data yield the accuracy. With the absolute value accounted for, the average accuracy is 90.66 %, 7.24 % lower than that of SVR.

**Table 7:** SVR vs. linear regression results.

Linear regression results				
	Prediction	Actual	Abs(deviation)	Accuracy
Maximum	22,489,961	26,346,522	3,856,561	99.32 %
Minimum	7,431,871	7,712,602	69,550	75.45 %
Average	15,713,040	16,185,923	1,490,350	90.67 %
Std Dev	3,676,309	4,110,707	978,310	6.38 %
SVR results				
	Prediction	Actual	Abs(deviation)	Accuracy
Maximum	23,853,917	26,346,522	949,877	99.99 %
Minimum	8,054,388	7,712,602	1534	95.38 %
Average	15,920,427	16,185,923	312,672	97.91 %
Std Dev	3,838,545	4,110,707	128,468	0.95 %

A comparison of predictive performance between SVR and multiple linear regression is conducted utilizing the root mean squared error (RMSE) and R-squared methods, commonly employed in statistical analysis. The outcomes affirm that the SVR prediction model outperforms the multiple linear regression method in terms of reliability and interpretative predictive capacity, as shown in Table 8. An essential factor influencing SVR prediction accuracy is the selection of the core function. Through grid search and cross-validation, samples collected in this study are trained and verified. Results indicate the radial basis function (RBF) is the optimal choice, yielding the highest training verification score. Additionally, the linear function model and polynomial Poly exhibit the lowest scores, as depicted in Table 9. In consideration of the sample, the RBF function is identified as the core function for the SVR model due to its capacity to effectively reflect complex nonlinear relationships between variables. Comparative analysis between the prediction errors of the SVR model and the multiple linear regression model further underscores SVR's efficacy. It is evident that SVR's prediction outperforms that of multiple linear regression. Comparative visualization of error distributions between these methods in Fig. 2 elucidates the differences: the error distribution for multiple linear regression features several values exceeding 2,500,000 NTD, with only a handful falling below 500,000 NTD. Conversely, SVR showcases greater stability, with its error distribution being predominantly below 3 %. This highlights the potential instability of multiple linear regression as compared to the more stable and accurate prediction capabilities of SVR. Moreover, a comparison is made between the prediction error of the SVR model and the multiple linear regression model as shown in Table 10.

**Table 8:** Comparison table of evaluation indicators.

Model type	Evaluation Index	
	RMSE	R^2^
SV	0.0989	0.9924
Multiple linear regression	0.5211	0.8091

**Table 9:** Comparison table of model training verification accuracy for different core functions.

Accuracy score	Core function		
	Radial basis function (RBF)	Linear (Linear)	Polynomial (Poly)
Training group average score	0.977238	0.769245	0.748012
Verified group average score	0.943653	0.753793	0.682164

**Table 10:** Comparison of prediction errors between SVR and multiple linear regression.

Predictive Model	Type of Error					
	Average absolute error	Maximum error	Minimum error	Average absolute percentage error	Maximum error percentage	Minimum error percentage
SVR	312,672	949,878	1533	2.0916	4.6178	0.0122
Multiple linear regression	1,490,349	3,856,561	69,549	9.3342	24.5530	0.6811

In summary, whether considering predictive accuracy or robustness, the SVR model consistently surpasses the multiple linear regression model. Comparative evaluations summarized in Table 4, Table 5, Table 6, Table 7 reinforce SVR's superior predictive potency and stability. This study, built on data from 238 EHV tower foundations, a typical small dataset, attests to SVR's exceptional prediction accuracy for such data. The SVR model can provide valuable assistance in future EHV transmission tower foundation reconstruction and expansion project planning. Its swift and accurate forecasting abilities make it pivotal for budget or reserve bidding price determinations, aiming to curtail Taipower contractors' estimated operational costs while enhancing administrative efficiency. Furthermore, it aims to improve the precision of annual budget allocation for the execution and reconstruction of EHV tower foundations.

## 6 Conclusion

This study has formulated an SVR model tailored to predict the construction costs of EHV transmission tower projects, specifically focusing on the expenditures associated with EHV Transmission tower foundations. The research was initiated by amassing data from 238 EHV transmission tower projects in Taiwan over a nearly decade-long span. Subsequently, the model is developed through the utilization of the Python programming language, integrated with machine learning techniques such as grid search and cross-validation. These methods are employed to identify the most fitting parameters for SVR across diverse core functions. The model encompasses three core functions: RBF, linear function (Linear), and Poly. These functions are introduced into the model for cross-validation training. The results emphasize that the SVR prediction model featuring RBF as the core function attains the highest training verification score, while the linear model is chosen, and the Poly model demonstrates the lowest score. This empirical finding corroborates the appropriateness of selecting RBF as the core function for the SVR prediction model. Comparative outcomes encompassing MAE, MAPE, error rates, maximum and minimum errors, RMSE, R2, and other verification metrics substantiate that the SVR model excels over the multiple linear regression model in terms of predictive strength and stability. Lastly, employing the developed SVR algorithm, EHV price forecasts are generated. The test data are incorporated into the forecasting model, resulting in forecasted outcomes that are juxtaposed with actual data. With a prediction accuracy of 97.9084 %, the SVR model demonstrates its capacity to effectively anticipate the costs associated with constructing ultra-high voltage tower foundations.

## Data availability

All data, models, and code generated or used during the study appear in the submitted article. Further discussion and data details of this study are available from the corresponding author upon reasonable request.

## CRediT authorship contribution statement

**Diana Wahyu Hayati:** Writing – original draft, Resources, Methodology, Investigation, Formal analysis, Data curation, Conceptualization. **Jieh-Haur Chen:** Writing – review & editing, Resources, Conceptualization, Data curation, Formal analysis, Funding acquisition, Investigation, Methodology, Project administration, Supervision. **Yu-Chun Chen:** Software, Resources, Project administration, Conceptualization. **Shixian Li:** Writing – review & editing, Software, Resources, Project administration, Conceptualization. **Machsus Machsus:** Visualization, Software, Resources, Methodology. **Mohamad Khoiri:** Validation, Software, Resources, Data curation. **Qian-Cheng Wang:** Writing – review & editing, Resources. **Hsi-Hsien Wei:** Writing – review & editing, Resources, Conceptualization.

## Declaration of competing interest

The authors declare that they have no known competing financial interests or personal relationships that could have appeared to influence the work reported in this paper.
